# Is noncoplanar plan more robust to inter‐fractional variations than coplanar plan in treating bilateral HN tumors with pencil‐beam scanning proton beams?

**DOI:** 10.1002/acm2.14186

**Published:** 2023-11-16

**Authors:** ByongYong Yi, Jenna Jatczak, Wei Deng, Yannick P. Poirier, Weiguang Yao, Matthew E. Witek, Jason K. Molitoris, Mark J. Zakhary, Baoshe Zhang, Nrusingh C. Biswal, Matthew J. Ferris, Sina Mossahebi

**Affiliations:** ^1^ Department of Radiation Oncology University of Maryland School of Medicine Baltimore Maryland USA; ^2^ Maryland Proton Treatment Center Baltimore Maryland USA

**Keywords:** head & neck, noncoplanar plan, proton PBS

## Abstract

**Purpose:**

Noncoplanar plans (NCPs) are commonly used for proton treatment of bilateral head and neck (HN) malignancies. NCP requires additional verification setup imaging between beams to correct residual errors of robotic couch motion, which increases imaging dose and total treatment time. This study compared the quality and robustness of NCPs with those of coplanar plans (CPs).

**Methods and Materials:**

Under an IRB‐approved study, CPs were created retrospectively for 10 bilateral HN patients previously treated with NCPs maintaining identical beam geometry of the original plan but excluding couch rotations. Plan robustness to the inter‐fractional variation (IV) of both plans was evaluated through the Dose Volume Histograms (DVH) of weekly quality assurance CT (QACT) sets (39 total). In addition, delivery efficiency for both plans was compared using total treatment time (TTT) and beam‐on time (BOT).

**Results:**

No significant differences in plan quality were observed in terms of clinical target volume (CTV) coverage (D95) or organ‐at‐risk (OAR) doses (*p* > 0.4 for all CTVs and OARs). No significant advantage of NCPs in the robustness to IV was found over CP, either. Changes in D95 of QA plans showed a linear correlation (slope = 1.006, R^2^ > 0.99) between NCP and CP for three CTV data points (CTV1, CTV2, and CTV3) in each QA plan (117 data points for 39 QA plans). NCPs showed significantly higher beam delivery time than CPs for TTT (539 ± 50 vs. 897 ± 142 s; *p* < 0.001); however, no significant differences were observed for BOT.

**Conclusion:**

NCPs are not more robust to IV than CPs when treating bilateral HN tumors with pencil‐beam scanning proton beams. CPs showed plan quality and robustness similar to NCPs while reduced treatment time (∼6 min). This suggests that CPs may be a more efficient planning technique for bilateral HN cancer proton therapy.

## 1 INTRODUCTION

Radiation therapy is often an essential component in head and neck (HN) cancer treatment. Proton therapy, specifically intensity‐modulated proton therapy (IMPT) using pencil‐beam scanning delivery techniques, has become a desirable way to deliver HN radiation because of dosimetric advantages over photon treatments, with corresponding clinical benefits that continue to be established.^1^, ^2^, ^3^, ^4^, ^5^, ^6^, ^7^, ^8^, ^9^

Noncoplanar plans (NCPs) are commonly used in treating HN cancer with IMPT, especially for the treatment of at‐risk lymph node regions in the bilateral neck.^10^, ^11^ Noncoplanar beams have often been utilized in an attempt to reduce doses to organs at risks (OARs) by effectively avoiding the OARs in the path of coplanar beams.^10^ In addition to OAR avoidance, superior‐oblique beams are expected to be more robust to daily setup (particularly shoulder position) variations, in cases where treatment extends to the lower neck region. However, NCPs have several disadvantages compared with coplanar plans (CPs) such as elongated treatment time and setup inconvenience.

The robotic couch used for modern proton treatments offers a convenient and efficient patient setup due to its six degrees of freedom (DOF) motion. The dosimetric effects of non‐robotic 6‐DOF couches in photon treatments have been well studied; however, only a few studies have reported table positional errors resulting from couch rotation in proton therapy.^12^, ^13^, ^14^, ^15^, ^16^, ^17^ An industrial robotic couch (KUKA, Germany) is used for the ProBeam (Varian, CA) proton treatment unit. It is reported that up to 4–20 mm displacement error in the horizontal plane when the couch rotates ±90°, which requires a relative isocenter shift correction.^18^ Biswal et al.^16^ demonstrated that orthogonal kV imaging is required between beams after couch rotation, as translational or angular shifts were necessary for more than 50% of noncoplanar HN cases. Treatment delivery time of high‐quality NCP is thus always longer than that of CP, since couch rotation and subsequent imaging take additional time. In addition, volumetric imaging or cone‐beam CT (CBCT) is not possible when the treatment couch is rotated, which makes for image‐guided radiation therapy that is somewhat inferior to that with CP.

As noted, the rationale behind NCPs in HN treatments is to provide more robust plans than CPs in terms of inter‐fractional variations (IV) due to daily setup differences or morphologic changes by arranging couch angles such that beams enter the patient from superior angles, farther from the shoulders. While intuitive, the assumption that this beam geometry leads to better plans has never been directly evaluated. With this study, we sought to compare the patterns of nominal plan quality variations, especially changes in target coverage between NCP and CP. In addition, we compared plan quality over the course of patient treatment via recalculating plans on quality assurance CTs (QACT).

## 2 METHODS

### 2.1 Patient selection and planning

In our Institutional Review Board–approved study, 10 patients with HN cancer who had previously received bilateral neck treatment using 4‐beam NCP were chosen by a random sampling method among the cases from September 2018 to August 2022 and were retrospectively planned with CP. All treatment plans were generated using the RayStation treatment planning system (V8‐V11A, RaySearch Lab; Stockholm, Sweden). Table 1 summarizes the treatment characteristics of the original NCP plans. Delineation of the target volume and OARs were performed in accordance with our institutional clinical practice guidelines and were unchanged between NCP and CP. Twenty‐one scenarios from the combination of seven positional uncertainty (no shift and ±3 mm of X, Y, and Z directions) and three range uncertainties (0% and ±3.5%) were used for the robust optimization and the robust evaluations. Treatment fields for all cases in Table 1 covered the entire neck region from the skull base or lateral process of the C1 vertebral body to the lower neck (at least to within 2 cm of the sternal notch; levels 2−4 ± 1B and 5 depending on the nuances of the case were covered). All cases were prescribed as simultaneous integrated boost (SIB) plans to treat three clinical target volumes (CTVs) in 30−35 fractions. High‐risk CTVs contained gross disease with small margins; intermediate‐risk CTVs contained intermediate‐risk elective areas, including portions of the ipsilateral neck; and low‐risk CTVs contained lower‐risk elective areas, including uninvolved contralateral neck, and occasionally, lower‐risk areas of the ipsilateral neck.

**TABLE 1:** Patient treatment characteristics.

Sl. no.	Gantry angles (°)	Couch angles (°)	# of QACT^a^	Total dose (cGy)
HN1	70, 290, 180.1, 0	345, 15	5^b^	5280, 5940, 6996
HN2	65, 295, 180.1, 0	345, 15	6^b^	5600, 6300, 7000
HN3	70, 290, 180.1, 0	340, 20	3	5600, 6300, 7000
HN4	75, 285, 180.1, 0	345, 15	3	5425, 6020, 7000
HN5	75, 285, 180.1, 0	345, 15	3	5600, 6300, 7000
HN6	70, 290, 180.1, 0	345, 15	5^b^	5100, 6000
HN7	70, 290, 180.1, 0	345, 15	4	5280, 6006, 6996
HN8	70, 290, 180.1, 0	345, 15	2	5280, 6006, 6996
HN9	70, 290, 180.1, 0	345, 15	5	5280, 6006, 6996
HN10	70, 290, 210, 150	340, 20	3	5100, 5400, 6300

^a^ Number of QACTs either for the entire treatment course (HN1, HN2, and HN 6) or before the replan (rest of the cases).

^b^ No Replans were needed.

**FIGURE 1:**
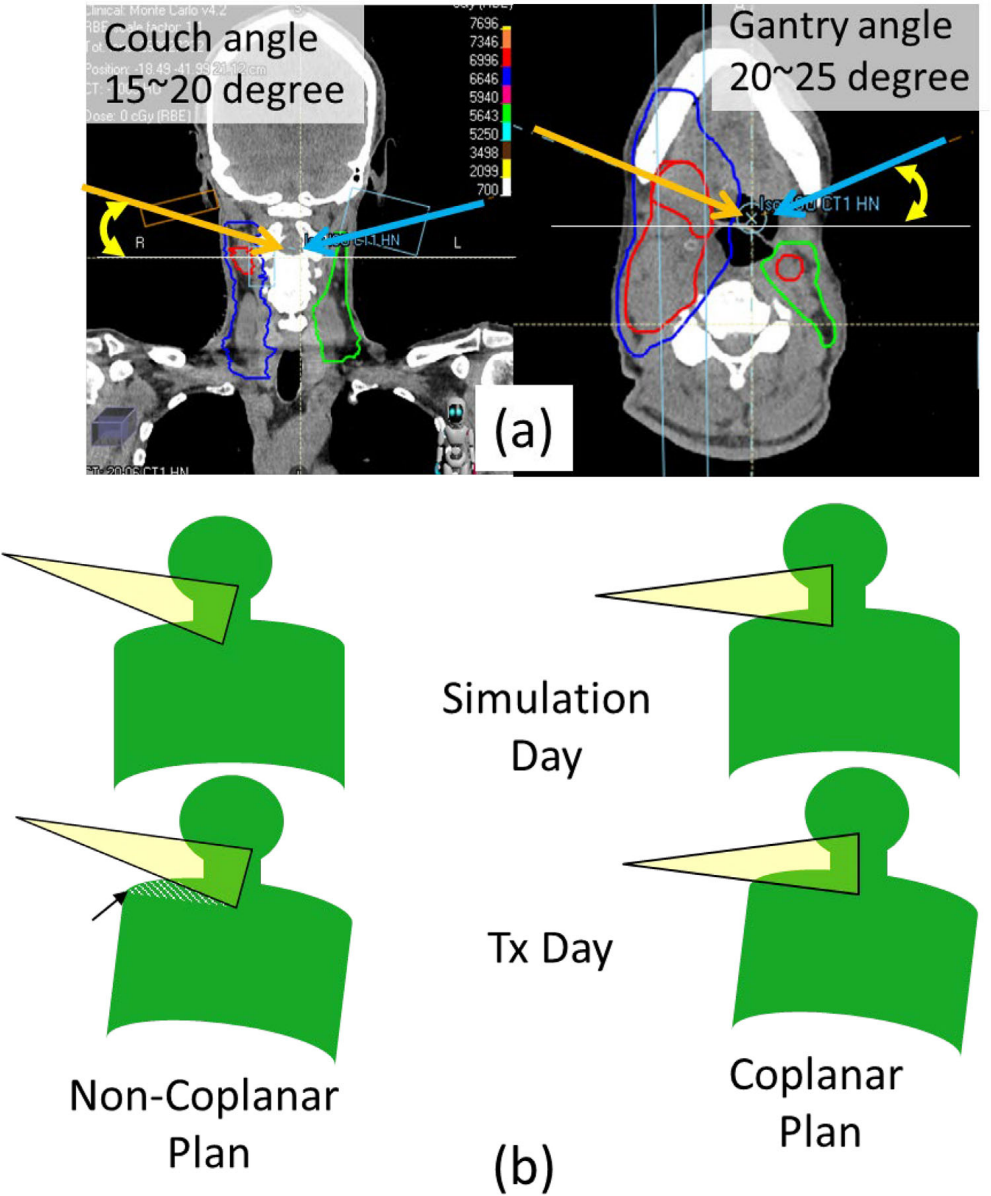
Beam alignment of clinical noncoplanar and competing coplanar plans. Couch angles of the noncoplanar beams were 15°−20°, whereas gantry angles were 15°−25° above the horizon (a). A noncoplanar beam is expected to be more robust to positional changes than a coplanar beam (b).

All originally planned NCPs used two coplanar and two noncoplanar beams. Orientations of the noncoplanar beams were superior–anterior obliques of both the left and right sides. Couch angles of noncoplanar fields were designed to be 15°−20°, with gantry angles anterior oblique at ∼15°−25°above the horizon (Figure 1). Each of the noncoplanar fields covered the ipsilateral side of the target and did not cover the contralateral side, in order to preserve the central nontarget region. Coplanar fields, typically at anterior and posterior angles, covered the lower neck (posterior field) and upper neck (anterior field) regions. Beam orientations, virtual beam blocking, and optimization constraints were arranged such that all target voxels received doses from two or more fields and none of the beams contributed >70% of the prescribed dose to any voxel, in order to maximize robust treatment delivery. Depending on the target shape and location, two posterior oblique fields were occasionally used instead of anteroposterior/posteroanterior fields (case HN10 in Table 1).

### 2.2 Plan comparisons

Rival CPs were generated with beam arrangements identical to those of the NCPs but excluding couch rotations. The nominal air gaps of the coplanar beams were maintained to be the same as those of noncoplanar beams. The same treatment planning constraint parameters as those for clinical NCPs were used for plan optimization. Plan quality parameters for CPs, such as target coverage as defined by D95% of all CTVs (CTV1, CTV2, and CTV3) and OAR doses for the oral cavity, parotid glands, larynx, and spinal cords, were compared with those from the clinically‐treated NCPs.

Weekly quality assurance (QA) CTs (QACTs) were acquired for all patients per institutional practice guidelines. QA plans (QPs) using the weekly QACTs were compared with initial plans (IPs) to monitor changes in plan parameters. Image registration and contour propagation of QACTs to planning CTs for dose evaluation were in accordance with our institutional clinical practice guidelines. To evaluate the plan robustness to different conditions, such as the setup variation or changes in morphology, patterns of the IV in plan qualities were compared between CPs and NCPs. Dose differences of D95 (DD‐D95) for CTVs between IP and QP were defined as D95 of QP subtracted by D95 of IP to determine dose degradation patterns between the two plan schemes.

Plan comparisons between IP and QP both for NCP and CP continued until each case was determined to have converted to a revised plan to accommodate variations or until the end of the treatment. Three patients maintained their IPs through their entire treatment courses, whereas seven patients converted to a revised plan as a result of unacceptable changes in plan parameters between IPs and QPs using routine QACT analysis. Ten IPs and 39 QPs (average, 3.9 per patient; range, 2−6) were used for plan comparisons. Of 39 QPs compared, seven were ultimately recommended for additional replanning.

### 2.3 Treatment time evaluation

Treatment times for the NCP cases shown in Table 1 were analyzed. Treatment times of seven fractions per case were used to rule out the effect of daily treatment delivery time variation of treatment machines. Total treatment time (TTT) was defined as the time from beam‐on of the first beam to beam‐off of the last beam. Initial patient setup time was not included in TTT. TTT was divided into two components: beam‐on time (BOT) and total beam waiting/imaging time between beams (TBB). Treatment times for another 10 patients with similar patient characteristics to those in Table 1 but treated with CPs were selected to determine treatment time differences between NCPs and CPs. The numbers of fields and beam angles were the same, except for couch rotations.

## 3 RESULTS

### 3.1 Comparisons of NCP and CP plan quality

**FIGURE 2:**
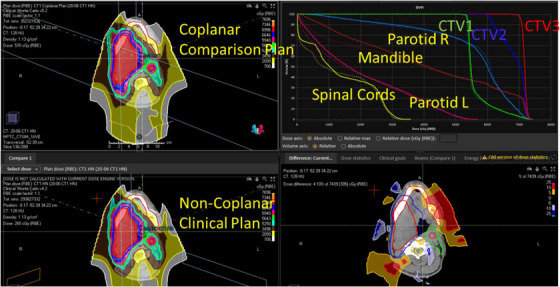
Plan comparisons between noncoplanar and coplanar plans for HN 1. The plan quality was almost the same. Solid lines are DVHs of CP, and dotted lines are those of NCP.

**FIGURE 3:**
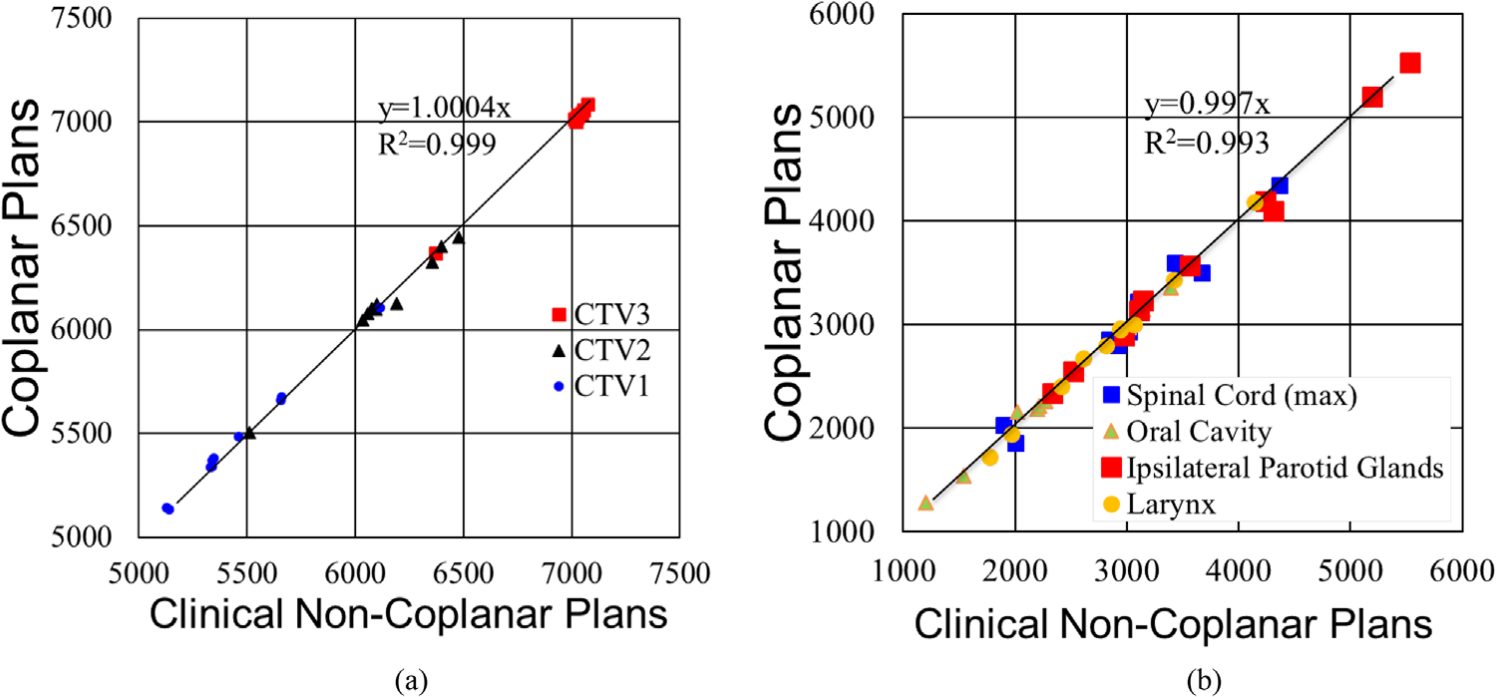
Relation between two plan schemes: (a) Target coverage (D95 of CTVs); and (b) OAR doses. OAR doses in (b) are mean doses except the spinal cord doses, which is the maximum dose. The Ipsilateral Parotid Glands in the figure is the mean dose to the parotid ipsilateral glands to gross disease.

Figure 2 shows a plan quality comparison between NCP and CP for case HN1 indicating almost identical dose distributions. Target coverage and OAR doses for all 10 cases were almost identical between the two sets of plans, as shown in Figure 3, with no significant difference found (*p* > 0.4). The slope and the coefficients of determination, R^2^ of the mean doses of OARs, (Figure 3b) were 0.997 and 0.993, respectively. D95 of all CTVs between NCP and CP also showed a linear relationship with the slope of 1.0004 and R^2^ > 0.999 (Figure 3a).

Medians of the worst‐case scenario of V95 coverage for 21 robust scenarios of NCP and CP planning schemes were 99 (max 98, min 96) and 99 (max 99, min 95) and no statistically significant difference was found between two plans (*p* = 0.8). A strong correlation (>0.999) was found between the two planning schemes of the dose of 1% of the CTV received (D1). The medians of D1 were 104.8% and 105.0% for NCP and CP, respectively. D1 difference in each case between the two planning schemes was <1%.

### 3.2 Comparisons of treatment delivery fidelity

**FIGURE 4:**
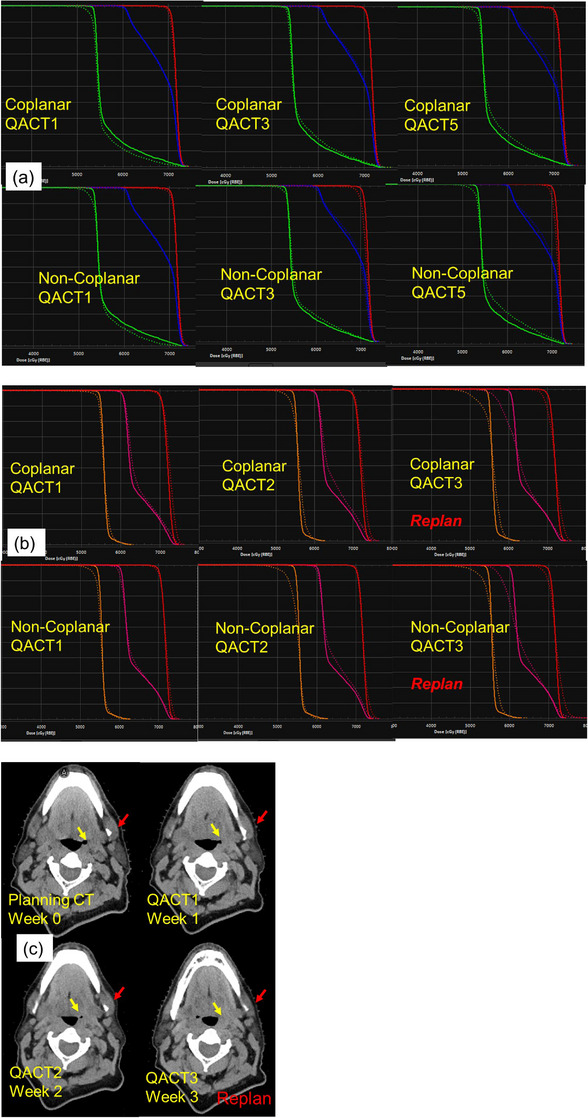
Comparisons of fractional variation of CTV DVHs between noncoplanar and coplanar plans for HN1, no replan case (a), and HN4, replan case (b and c). Patterns of change of DVHs were almost identical between NCP and CP. Solid lines are DVHs of the initial plan, and dotted lines are those of QA plan of that week.

Figure 4a shows the DVHs for 5 weeks with the two schemes of plans for case HN1, for which no replan was required. Neither of the planning schemes demonstrated a clinical need to replan based on evaluation QPs. On the other hand, a replan was needed for case HN4 at the third QP, as shown in Figure 4b. For case HN4, the CTV1 DD‐D95 at week 2 started to increase notably. At week 3, the DD‐D95 of CTV1 and CTV2 increased significantly. It was therefore determined to use a new plan to adapt to the anatomic changes. The region indicated by the yellow arrow Figure 4b began to change from week 2, and the red arrow–denoted region started to change from week 3. Both NCP and CP planning schemes indicated the need for a revised plan at week 3. Cases HN1 and HN4 both show that NCPs were not necessarily more robust to anatomic variations or to fractional setup variations that may have existed. Neither Figure 4a nor 4b show noticeable DVH differences between NCPs and CPs.

**FIGURE 5:**
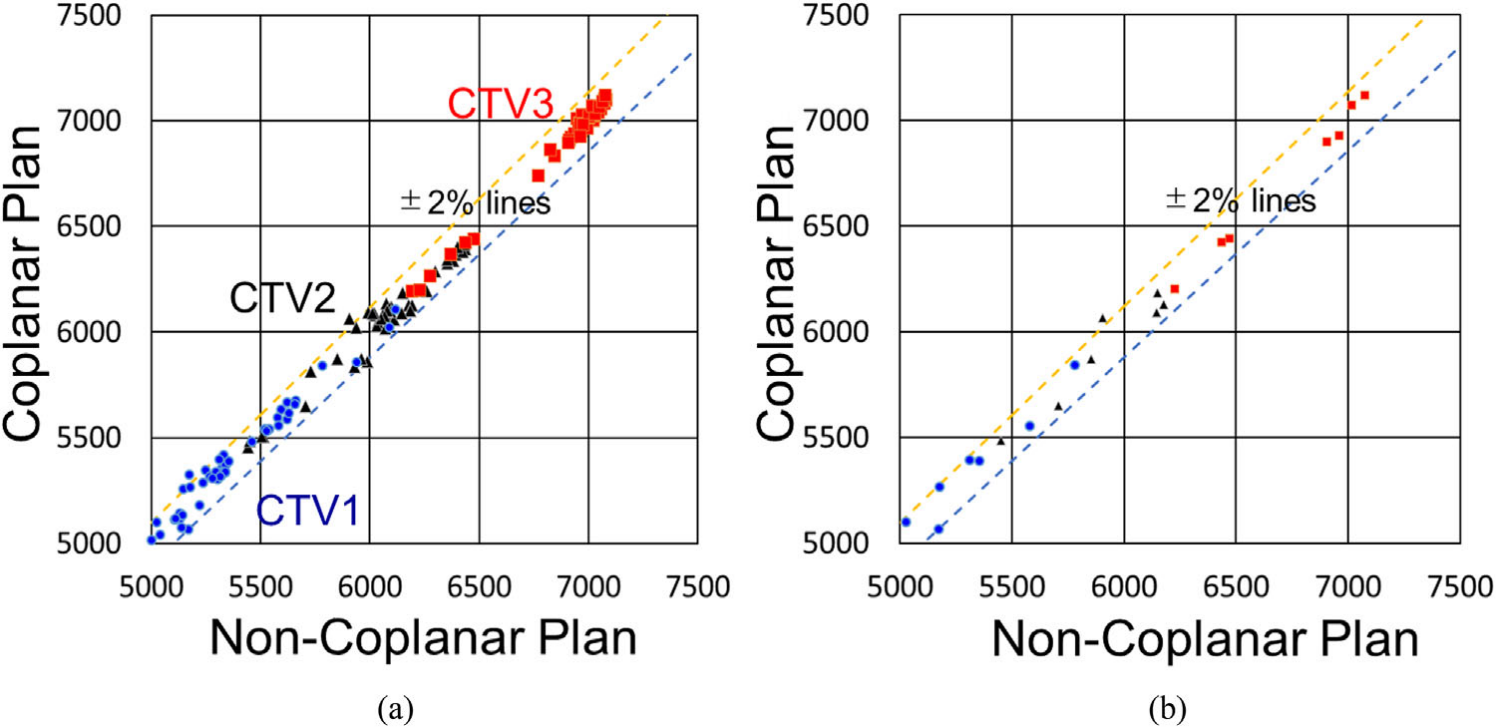
Fractional dose variation of D95 of each CTV for (a) all cases and (b) replan cases only. No case showed *a* > 3% differences between NCP and CP. All cases included CTV1, CTV2, and CTV3 of replan cases and no‐replan cases. Differences between NCP and CP were <2% for >95% of data points.

As demonstrated in Figure 5 for all cases and replan cases, the slopes of D95 for CP and for NCP were almost unity: 1.006 and 1.008, respectively. Both of the coefficients of determination, R^2^_,_ were >0.99. Dose differences between CP and NCP (Figure 5) were <2% for >95% of the data points. Equivalence tests of the variation of D95 for each QP between the two planning strategies, CP and NCP, using two one‐sided tests (TOST) showed statistical equivalence (the larger of the two *p* values of the upper and the lower equivalence bounds was *p* < 0.0001, within 95% confidence interval). Equivalence bounds were chosen to be ±2% of D95 of NCP IP. TOST of the replan cases also showed statistical equivalence (*p* < 0.0001).

### 3.3 Treatment time

Table 2 shows the monitor units (MUs) and BOTs for both groups. There were no significant differences noted between total MUs (76,262 ± 13,389 for CPs; 76,011 ± 16,319 for NCPs; *p* = 0.36) or BOTs (271 ± 35 s for CPs; 283 ± 59 s for NCPs; *p* = 0.35) between the two groups. Table 3 shows the treatment times for both groups. Both TTTs (539 ± 50 s for CP; 897 ± 142 s for NCP; *p* < 0.001) and TBBs (269 ± 44 s for CP; 614 ± 121 s for NCP; *p* < 0.001) were significantly different between the two groups. TTTs were 6 min shorter per patient for CPs than NCPs.

**TABLE 2:** Comparison of beam‐on time and monitor units (MUs) for coplanar planning (CP) and noncoplanar planning (NCP) groups.

	Beam‐on time (s)		Total monitor units (MUs)	
	CP	NCP	CP	NCP
Time/MU	271 ± 35	283 ± 59	76,262 ± 13,389	76,011 ± 16,319
Difference	12		−251	
*p*	0.35		0.36	

**TABLE 3:** Comparison of total treatment time and time between beams between coplanar planning (CP) and noncoplanar planning (NCP) groups.

	Time between beams (s)		Total treatment time (s)	
	CP	NCP	CP	NCP
Time	269 ± 44	614 ± 121	539 ± 50	897 ± 142
Difference	345		358	
*p*	<0.001		<0.001	

## 4 DISCUSSION

The quality of treatment delivery changes for multiple reasons throughout the course of radiotherapy treatments, especially as a result of anatomic changes and daily setup variations. It may seem intuitive that a noncoplanar approach with lateral beams entering from superior directions would be more robust against daily setup variations, especially inter‐fractional shoulder motions as illustrated in Figure 1b. However, if this assumption were valid, we would observe more dose variations between the IPs and QPs of CPs than those of NCPs.

This study consists of two steps. The first step is to prove the plan qualities of CPs are not worse than those of NCPs. The second step is to show the patterns of replans due to IV are the same between the two schemes. In other words, this study compared IP quality parameters and temporal changes in plan qualities between CP and NCP. It is important to note that causes of the IV of the delivered dose distributions from IP to QA, consist of not only the daily setup differences but also patient's anatomical changes, which can also cause treatment delivery to deviate from IPs, regardless of planning schemes. The first step was tested as follows. Both D95 of CTVs and OAR doses showed linear relationships with a slope of one between the two planning schemes, indicating that their plan quality parameters were identical and unchanged by planning methodology. The worst‐case scenario comparisons of the plan robustness tests showed no statistical difference between CP and NCP plans and D1 of the two planning approaches showed no statistically significant difference but a strong correlation. The second step compared the temporal plan quality variations. The slopes of temporal plan quality variations between the two schemes for CTVs and OARs were 1 ± 0.05 and R^2^ > 0.99. If treatment delivery was more robust for one planning scheme than the other, we would instead observe a non‐unity slope and/or less correlation (R^2^ < 0.8). Dose differences between CP and NCP of D95 were <2% for >95% of the data points. TOST equivalent tests for all cases and for replan cases showed that D95 variations for NCP and CP were statistically identical. These results suggest that NCP is not more robust than CP to setup variation or to morphologic changes.

Treatment delivery of NCP inherently requires more attention paid to patient safety which increases treatment time. Biswal et al.^16^ demonstrated that extra setup verification images are required when the robotic couch is rotated from the analysis of their 28 patients. Of the 3219 Cartesian shifts, 2069 (64.3 %) were zero and 1150 (35.7 %) were non‐zero (range, −7 to 11 mm). Of the 2146 angular shifts, 1034 (48.2) were zero and 1112 (51.8 %) were non‐zero (−3.0° to 3.2°). The setup variations observed in this research were consistent with Biswal's study. A total of 888 Cartesian and 592 angular shift values were recorded for 10 patients, during the whole course of treatments. Of the Cartesian shifts, 542 were zero (61%) and 346 (39%) were non‐zero (range, −6 to 6 mm). Of the angular shifts, 278 (47 %) were zero and 314 (53%) were non‐zero (range, −2.0° to 2.5°).

In determining TTT, initial setup time was excluded, because initial setup times are independent of the treatment technique, unless the first treatment field delivered happens to be a noncoplanar beam. Beam waiting time between treatment rooms may vary because of concurrent activities in other treatment rooms. In order to mitigate spurious data fluctuation from this irrelevant external effect, treatment times were collected over seven fractions for each patient. On average, CP treatment times were 358 s (average) shorter than those for NCP, due to increased setup time between beams and extra imaging time in non‐coplanar beams. The use of CBCT images in setup is useful for soft tissue‐based setup. It also is useful for reducing the frequency of the quality assurance CT for HN proton treatments.^11^ We often lose these advantages of CBCT since the acquisition of CBCT is not possible when the couch is rotated. Additionally, the use of noncoplanar beams generates another challenge as Lin et al.^19^ noted a limitation in using NCP with their universal patient‐related range shifter as a result of potential collision. In this study, either the nominal air gaps or the minimum air gaps between the treatment nozzle and patients’ skin surface were not noticeably different for two reasons: (1) The nominal air gap of CP was set to be the same of that of NCP; and (2) Since the beam angles are 15°–20° above the horizon, the chances of collisions are not much different between two planning schemes.

Despite the advantages of CP as noted here, there is still a potential risk that the beam path passes through a significant part of the shoulder when using this planning methodology (Figure 1b). To avoid this, we recommend avoiding using lateral gantry angles (90° or 270°) and instead using introducing a ± 15° shift or more from the horizontal directions. If a case requires gantry angles to be in a range within 90° (or 270°) ± 15°, then NCP remains superior. Gantry angles in all cases in Table 1 utilized gantry angles of ±15° or more from the horizontal directions.

## 5 CONCLUSION

Plan qualities were almost identical between NCP and CP in terms of CTV coverage and OAR doses in treating bilateral HN cancer patients with proton PBS. No evidence was found that NCP was more robust than CP in terms of IV. On the other hands, CP significantly reduced treatment times and imaging doses while providing similar plan quality. CP is the preferred approach for bilateral HN cancer treatment with proton therapy.

## AUTHOR CONTRIBUTIONS

The authors confirm contributions to the paper as follows: **study conception and design**: ByongYong Yi; Matthew Witek; Jason K. Molitoris. **Data acquisition**: ByongYong Yi; Jenna Jatczak; Sina Mossahebi. **Analysis and interpretation of results**: ByongYong Yi; Wei Deng; Yannick P. Poirier; Weiguang Yao; Matthew Witek; Jason K. Molitoris; Mark J. Zakhary; Baoshe Zhang; Nrusingh Biswal; Matthew J. Ferris; Sina Mossahebi.

## CONFLICT OF INTEREST STATEMENT

The authors declare no conflicts of interest.

## Data Availability

Data that support the findings of this study are available from the corresponding author upon reasonable request.
